# Proposal for improving the system of physical therapy education and the Korean physical therapist licensing examination based on a comparison of the systems in World Confederation for Physical Therapy member countries

**DOI:** 10.3352/jeehp.2017.14.10

**Published:** 2017-05-24

**Authors:** Min-Hyeok Kang, Tae-Hwan Lee, Sang-Min Cha, Jae-Seop Oh, Tae-Sik Lee, Tae-Young Oh, Suhn-Yeop Kim, Hyoung-Soo Lee, Gyu-Wan Lee, Ki-Song Kim

**Affiliations:** 1 Department of Physical Therapy, International University of Korea, Jinju, Korea; 2 Department of Physical Therapy, Graduate School, Inje University, Gimhae, Korea; 3 Department of Physical Therapy, College of Biomedical Science and Engineering, Inje University, Gimhae, Korea; 4 Department of Physical Therapy, Dong-Eui Institute of Technology University, Busan, Korea; 5 Department of Physical Therapy, Medical Life Science College, Silla University, Busan, Korea; 6 Department of Physical Therapy, College of Health and Medical Science, Daejeon University, Daejeon, Korea; 7 Department of Physical Therapy, Gwangju Health University, Gwangju, Korea; 8 Department of Physical Therapy, Gangnam Severance Hospital, Yonsei University Health System, Seoul, Korea; 9 Department of Physical Therapy, Life and Health Science College, Hoseo University, Asan, Korea; Hallym University, Korea

The process of nurturing and producing health and medical service personnel, including physical therapists, comprises 3 stages: training provided by educational institutions, evaluation for qualification, and a management system after certification. Therefore, we needed to review the physical therapy programs in Korea and the subjects covered by the Korean physical therapist licensing examination as a first step in determining strategies for producing physical therapists with a high degree of knowledge and expertise. The first official physical therapy education department was established in a junior college of medicine attached to Korea University in 1963. Subsequently, 2-year college physical therapy courses were established at Shingu College, Dongnam Health College, Wonkwang Health Science College, Daegu Health College, and Kwangju Health College [1]. In 1979, Yonsei University established the nation’s first 4-year physical therapy educational program. In 1991, physical therapy programs were changed from a minimum of 2- to 3-year programs, increasing the number of courses and clinical training hours that students were required to complete [1]. As of 2016, physical therapy departments have been established in 87 colleges and universities across Korea [2]; during their undergraduate studies, students majoring in physical therapy take courses in foundations of physical therapy; physical therapy diagnosis, examination, and evaluation; physical therapy intervention; and medical law. Although the role of physical therapists has become even more important and specialized around the world, no significant changes have been made in physical therapy educational programs in Korea except for their duration. No standardization policies to establish a specific curriculum have been implemented. Therefore, we aimed to make proposals for improving physical therapist education in Korea by comparing physical therapy curricula, graduation degrees, and the allocation of subjects of items included in the Korean physical therapist licensing exams.

To analyze physical therapy school systems and graduation degrees in Korea and abroad, the colleges and universities listed on the Korean Physical Therapy Association website were examined. Based on the results of this investigation, we divided the schools into 2 categories: 3-year college programs and 4-year university programs [1]. The World Confederation for Physical Therapy (WCPT) and the Organization for Economic Cooperation and Development (OECD) member countries were reviewed to compare and analyze both domestic and foreign physical therapy education courses and school systems. Information on each country’s school system was gathered based on data provided by the WCPT. Of the 113 WCPT member countries, 34 countries are OECD member countries [2,3]. However, the WCPT refused to provide information on 3 of the OECD member countries; therefore, we examined only 31 countries in total.

Turning to the physical therapist licensing examination in Korea and the United States, we classified the subjects covered on the Korean physical therapist licensing examination and examined the percentage of items for each subject to compare domestic and foreign licensing examinations. The subjects in the Korean physical therapist licensing examination included foundations of physical therapy; physical therapy diagnosis, examination, and evaluation; physical therapy intervention; and medical law (subjects involving practical tests were excluded) [4]. The percentages of items per subject were analyzed. We then examined the subjects covered in the physical therapist licensing examination in the United States, which boasts advanced health and medical services. The US examination included 5 subjects: physical therapy examination; foundation for evaluation, differential diagnosis, and prognosis; intervention; equipment, devices, and therapeutic modalities; and safety and protection, professional responsibilities, and research [5]. The percentages of items for each subject were based on the examination results.

All data, including physical therapy curricula, graduation degrees, and the subjects of the physical therapists licensing examination, were searched from February 2016 to December 2016.

Departments of physical therapy have been established in 39 colleges (education period of 3 years) and 48 universities (education period of 4 years) in Korea. The average education period in these programs was 3.6 years. The longest physical therapist education period was 4 years for WCPT and OECD member countries (38.7%). The average education period for these countries was 3.8 years, longer than that in Korea (Table 1). While the minimum graduation degree for physical therapy students in Korea was a diploma, the largest share (67.7%) was the bachelor’s degree in both WCPT and OECD member countries (Table 2). When we looked at the percentage of physical therapist licensing examination items in Korea, physical therapy intervention took the largest share (35.0%), followed by foundations of physical therapy (30.0%); physical therapy diagnosis, examination, and evaluation (25.0%); and medical law (20.0%) (Table 3). In contrast, in the United States, physical therapy diagnosis, examination, and evaluation accounted for 59.0% of the total items, more than physical therapy intervention (28.5%); equipment, devices, and therapeutic modalities (6.0%); and safety and protection, professional responsibilities, and research (6.5%) (Table 4).

The above results show that the physical therapy education period in Korea is slightly shorter than that in other countries, and that the percentage of examination, evaluation, and diagnosis items on the licensing examination was smaller on the Korean physical therapist licensing examination than on the US examination. Efforts have been made for the past 20 years in the United States to award doctoral degrees to physical therapists (doctor of physical therapy), similar to the degrees held by medical doctors and pharmacists [6]. However, physical therapy education in Korea is provided through both 3- and 4-year programs; there is no distinction between these types of programs in terms of requirements for taking the licensing examination, and there is no significant difference in the educational programs themselves. The American Physical Therapy Association announced a “Vision Statement 2020,” stating that “By 2020, physical therapy will be provided by doctors of physical therapy, and recognized by clients and other experts as the practices which include direct access for the diagnosis, therapeutic interventions, and prevention of impairments, activity limitations and other restrictions in health” [7]. Considering the changes in the educational systems for physical therapists in the United States and other foreign countries, we believe that the educational system for physical therapy in Korea should be unified into a 4-year program and developed into a doctoral course according to social demands to nurture and produce physical therapists with a high level of knowledge and expertise.

Evaluation and diagnosis accounted for the largest share of items on the US physical therapist licensing examination, while interventions accounted for the largest share on the Korean licensing examination. Physical therapist licensing examinations should include content regarding all aspects of the actual jobs of these professionals, given that licensing examinations for health and medical service personnel should be designed to evaluate their expertise and professional abilities. According to the results of previous studies, diagnosis, measurement, and evaluation by physical therapists, as well as clinical decision-making, were tasks of high importance and frequency; diagnosis and evaluation were found to have higher importance and frequency than interventions [8]. Diagnosis and evaluation of a patient’s symptoms and condition are important elements in a physical therapist’s determination of whether they can handle a particular disease and/or provide an effective intervention. In physical therapist curricula in US universities, credits for subjects regarding evaluation, differential diagnosis, and foundations of prognosis accounted for larger shares of total credits than those related to physical therapy interventions [8]. Therefore, to nurture and produce excellent physical therapists, the proportion of items regarding diagnosis and evaluation should be re-allocated so that the actual performance and ability of physical therapists can be evaluated.

There are some limitations in this analysis. First, it does not suggest how to unify the current physical therapist educational system into a 4-year program or how to improve educational programs. Future studies will need to provide strategies to unify the current system into a 4-year program. Moreover, we only compared physical therapist licensing examination subjects between Korea and the United States; thus, the licensing examination systems of other OECD member countries need to be analyzed in future studies.

In conclusion, we propose that physical therapist educational programs in Korea should be unified into a 4-year program, and that the percentage of diagnosis and evaluation items should be re-allocated in the Korean physical therapist licensing examination. We believe that the results of this study will help to improve the quality of physical therapists graduating from educational programs in Korea.

## Figures and Tables

**Table 1.** Education period for physical therapists in World Confederation for Physical Therapy and Organization for Economic Cooperation and Development member countries in 2016

Education period (yr)	Countries	No. of countries (%)
3	Norway, Germany, Sweden, Slovakia, Slovenia, Estonia, United Kingdom, Austria, Italy, Czech Republic	10 (32.26)
3.5	Denmark, Finland	2 (6.45)
3-4	Japan, France, Korea (South)	3 (9.67)
4	Netherlands, New Zealand, Mexico, Switzerland, Spain, Iceland, Ireland, Israel, Turkey, Portugal, Hungary, Australia	12 (38.71)
4-5	Belgium	1 (3.23)
5	Chile	1 (3.23)
6	Canada, United States	2 (6.45)

**Table 2.** Minimum graduation degree for physical therapists in World Confederation for Physical Therapy and Organization for Economic Cooperation and Development member countries in 2016

Minimum graduation degree	Countries	No. of countries (%)
Diploma	Germany, Slovakia, Estonia, japan, France, Korea (South)	6 (19.35)
Bachelor's degree	Netherlands, Norway, New Zealand, Denmark, Mexico, Sweden, Switzerland, Spain, Slovenia, Iceland, Ireland, United Kingdom, Austria, Israel, Italy, Czech Republic, Chile, Turkey, Finland, Hungary, Australia	21 (67.74)
Graduate diploma	Portuga	1 (3.23)
Master's degree	Belgium, Canada	2 (6.45)
Professional doctorate	United States	1 (3.23)

**Table 3.** Subjects and the number of items per subject on the Korean physical therapist licensing examination

Subjects	Specific topic	No. of total items (%)
Foundations of physical therapy	Anatomy, biomechanics, physical agents, public health	60 (30)
Physical therapy diagnosis, examination, and evaluation	Measurement and evaluation, physical therapy diagnosis, clinical decision making, physical therapy problem-solving (diagnosis and evaluation)	50 (25)
Physical therapy interventions	Physical therapy for musculoskeletal problems, physical therapy for neurologic problems, physical therapy for cardiovascular and pulmonary problems, physical therapy for integumentary problems, physical therapy problem-solving (intervention)	70 (35)
Medical regulation	Medical regulation	20 (10)

**Table 4.** Subjects and the number of items per subject on the US physical therapist licensing examination

Subjects	Specific topic	No. of total items (%)
Physical therapy examination (26.50%)	Musculoskeletal	22 (11.00)
	Neuromuscular	17 (8.50)
	Cardiopulmonary	10 (5.00)
	Other system	4 (2.00)
Foundation for evaluation, differential diagnosis, and prognosis (32.50%)	Musculoskeletal	18 (9.00)
	Neuromuscular	15 (7.50)
	Cardiopulmonary	12 (6.00)
	Other system	20 (10.00)
Intervention (28.50%)	Musculoskeletal	21 (10.50)
	Neuromuscular	18 (9.00)
	Cardiopulmonary	11 (5.50)
	Other system	7 (3.50)
Equipment and device; therapeutic modalities (6.00%)	Equipment and device	5 (2.50)
	Therapeutic modalities	7 (3.50)
Safety and protection; professional responsibilities; research (6.50%)	Safety and protection	5 (2.50)
	Professional responsibilities	4 (2.00)
	Research	4 (2.00)

## References

[1] Korean Physical Therapy Association (2016). National university [Internet]. http://www.kpta.co.kr/kpta/#/about/university?category=10.

[2] Organization for Economic Cooperation and Development (2016). Countries [Internet]. http://www.oecd.org/about/membersandpartners/.

[3] World Confederation for Physical Therapy (2016). Member organizations [Internet]. http://www.wcpt.org/members.

[4] Department of Education of Pacific Books (2015). Tank manual of physical therapy: physical therapy.

[5] Giles SM (2014). PTEXAM: the complete study guide.

[6] McKinnis LN (2014). Fundamentals of musculoskeletal imaging.

[7] American Physical Therapy Association (2016). Vision 2020 [Internet]. http://www.apta.org/Vision2020/.

[8] Kang MH, Kwon OY, Kim YW, Kim JW, Kim TH, Oh TY, Weon JH, Lee TS, Oh JS (2016). Is there an agreement among the items of the Korean physical therapist licensing examination, learning objectives of class subjects, and physical therapists’ job descriptions?. J Educ Eval Health Prof.

